# Successful birth after preimplantation genetic testing for a couple with two different reciprocal translocations and review of the literature

**DOI:** 10.1186/s12958-021-00731-2

**Published:** 2021-04-20

**Authors:** Dun Liu, Chuangqi Chen, Xiqian Zhang, Mei Dong, Tianwen He, Yunqiao Dong, Jian Lu, Lihua Yu, Chuanchun Yang, Fenghua Liu

**Affiliations:** 1 Reproductive Medical Center, Guangdong Women and Children Hospital, Guangzhou, Guangdong China; 2 Medical Genetic Centre, Guangdong Women and Children Hospital, Guangzhou, Guangdong China; 3 CheerLand Precision Biomed Co., Ltd., Shenzhen, Guangdong China

**Keywords:** Reciprocal translocations, PGT-SR, Genetic counseling, Translocation breakpoints, Whole genome low-coverage sequencing

## Abstract

**Background:**

Preimplantation genetic testing for chromosomal structural rearrangements (PGT-SR) is widely applied in couples with single reciprocal translocation to increase the chance for a healthy live birth. However, limited knowledge is known on the data of PGT-SR when both parents have a reciprocal translocation. Here, we for the first time present a rare instance of PGT-SR for a non-consanguineous couple in which both parents carried an independent balanced reciprocal translocation and show how relevant genetic counseling data can be generated.

**Methods:**

The precise translocation breakpoints were identified by whole genome low-coverage sequencing (WGLCS) and Sanger sequencing. Next-generation sequencing (NGS) combining with breakpoint-specific polymerase chain reaction (PCR) was used to define 24-chromosome and the carrier status of the euploid embryos.

**Results:**

Surprisingly, 2 out of 3 day-5 blastocysts were found to be balanced for maternal reciprocal translocation while being normal for paternal translocation and thus transferable. The transferable embryo rate was significantly higher than that which would be expected theoretically. Transfer of one balanced embryo resulted in the birth of a healthy boy.

**Conclusion(s):**

Our data of PGT-SR together with a systematic review of the literature should help in providing couples carrying two different reciprocal translocations undergoing PGT-SR with more appropriate genetic counseling.

## Background

Balanced reciprocal translocations, an exchange of two terminal segments from different chromosomes, occur in approximately one in every 500–625 human newborns [1]. Carriers of reciprocal translocations usually have a normal phenotype, except when the translocation breakpoint results in gene interruption. Nevertheless, in most cases, these individuals are at high risk of producing unbalanced gametes, which associate with infertility, recurrent pregnancy loss or offspring abnormality [2, 3]. For reciprocal translocations, unbalanced gametes are likely to be generated owing to abnormal segregation patterns at meiosis.

During meiosis, three theoretical segregation patterns (2:2, 3:1 or 4:0) might occur in the presence of a reciprocal translocation, resulting in 32 possible gametes with the consideration of recombination [4]. But only two gametes from the alternate segregation mode are normal or balanced, and the others are unbalanced with an estimated prevalence of 60–70% [4–6]. However, Preimplantation genetic testing for chromosomal structural rearrangements (PGT-SR) following an in vitro fertilization (IVF) procedure has become an attractive option for translocation carrier couples to improve the pregnancy outcomes by selecting balanced/euploid embryos [1, 7]. To date, the vast majority of the PGT-SR studies were conducted in couples in which one of the partners is a carrier for a reciprocal translocation [2, 4–8]. By contrast, limited knowledge is known on the data of PGT-SR when both parents have a reciprocal translocation. Are double translocations associated with double risks? Here, we for the first time present a healthy live birth derived from a non-consanguineous couple carrying two different reciprocal translocations involving four chromosomes by PGT-SR combine with translocation breakpoint identification and show how relevant genetic counseling data can be generated. Moreover, we specifically reviewed the available literature to estimate the reproductive risk and discuss counseling approaches when couples with double reciprocal translocations.

## Methods

### Case presentation

**Fig. 1:**
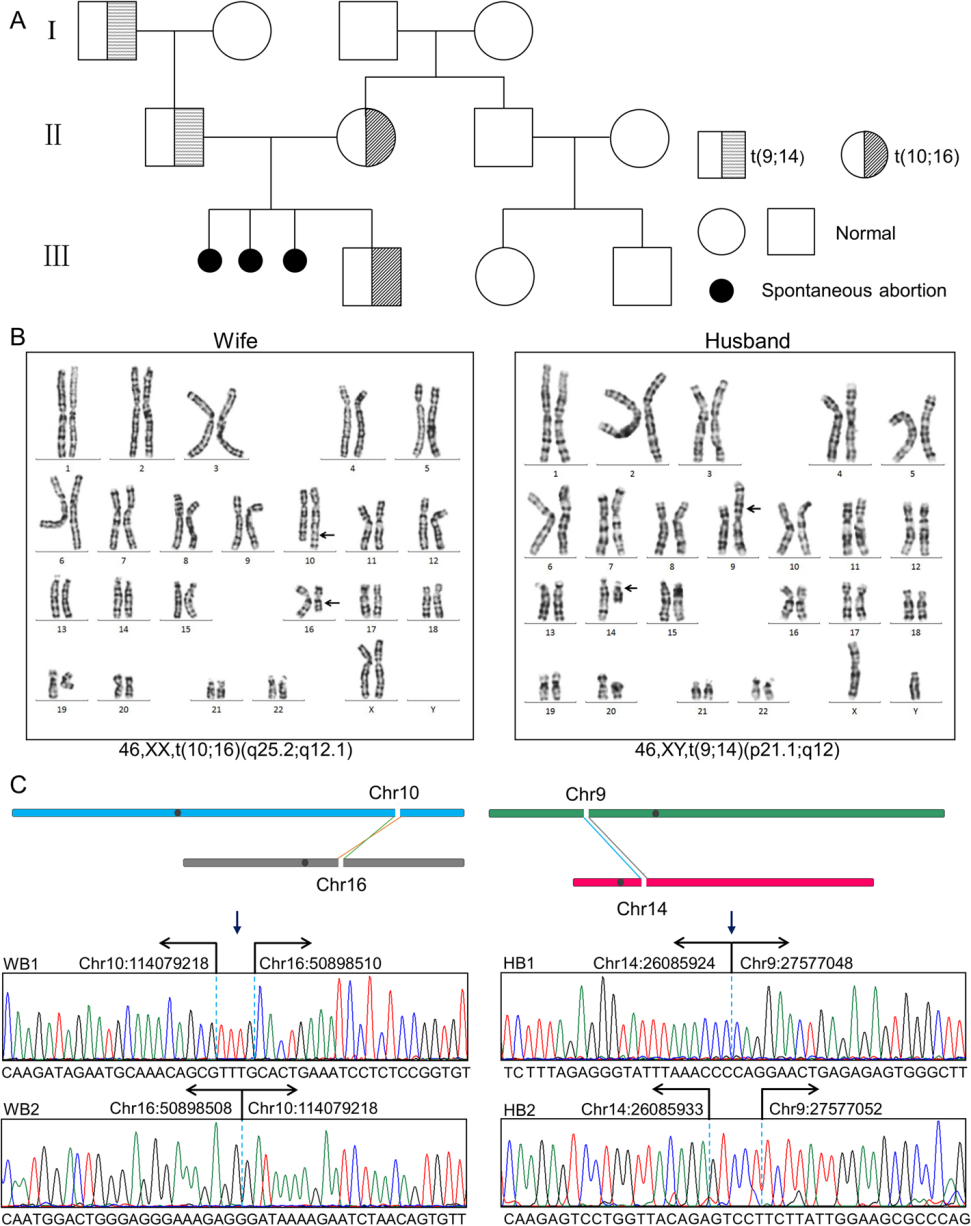
Pedigree study and translocation breakpoint identification for the couple. **a** Pedigree of the family. **b** Karyotype analysis of the wife 46,XX,t(10;16)(q25.2;q12.1) and the husband 46,XY,t(9;14)(p21.1;q12). **c** Junction-spanning PCR with Sanger sequencing showed four precise reciprocal translocation breakpoints of the couple. WB1 = wife breakpoint 1; WB2 = wife breakpoint 2; HB1 = husband breakpoint 1; HB2 = husband breakpoint 2

A 30-year-old woman and her 35-year-old husband, both phenotypically normal, were not consanguineous. They were referred for a 5-year history of secondary infertility and had experienced 3 consecutive spontaneous abortions at 5 or 6 weeks of gestation, none of which had been cytogenetically examined. The wife’s gynecological examination was normal and the husband had no abnormality on semen analysis. No histories of abnormal pregnancy were reported in the family history, as shown in the pedigree (Fig. 1a).

This study was reviewed and approved by the Institutional Review Board (IRB) of Guangdong Women and Children Hospital. Written consent was obtained from parents before commencing the study.

### Cytogenetic study

Cytogenetic karyotype analyses were performed on peripheral blood lymphocytes from the couple according to the conventional technique.

### Identification of precise translocation breakpoint

Genomic DNA was extracted from peripheral blood using the standard phenol/chloroform method. To analyses the molecular karyotype of the couple, we applied whole genome low-coverage sequencing (WGLCS) to initially identify the four breakpoint regions of the reciprocal translocations. The details of this method can be found in our previous report [9]. In brief, approximately 500 ng genomic DNA samples from translocation carriers were sheared into small (~ 500 bp) fragments for the small-insert library construction. Then, the genomic libraries were subjected to 50-bp-end multiplex sequencing on the Illumina HiSeq TM 2000 platform. For bioinformatics analysis, high-quality paired-end sequencing reads were aligned to the National Center for Biotechnology Information human reference genome (hg19, GRCh37.1) using SOAP2 [10] with parameters that include the total allowed mismatches (−*v* 2), seed length (−*s* 35), minimal aligning length (−*l* 23), and insert DNA size ranging from 400 to 600 bp. Only unique reads were retained for further analysis. The chimeric read pairs would suggest the possible candidate translocation “clusters” throughout the genome through data filtering [9]. The sequences included in the flanking region of the putative breakpoint regions were verified using polymerase chain reaction (PCR) with junction-spanning primers, followed by subsequent identification of the precise position of the breakpoints through Sanger sequencing.

### Controlled ovarian stimulation and in vitro fertilization (IVF)

Controlled ovarian stimulation was performed using gonadotropin-releasing hormone (GnRH) (Merck KGaA, Darmstadt, Germany) agonist, recombinant follicular-stimulating hormone (FSH) (Merck KGaA, Darmstadt, Germany) and human chorionic gonadotropin (HCG) (Livzon, Zhuhai, China). Standard techniques were used in IVF treatment, including fertilization, embryo culture, blastocyst biopsy, and blastocyst transfer at the Reproductive Medical Centre of Guangdong Women and Children Hospital.

### PGT-SR and embryo carrier testing

Biopsied trophectoderm (TE) cells for PGT-SR were used for whole-genome amplification (WGA) using the PicoPLEX single-cell WGA kit (Rubicon Genomics, Ann Arbor, USA). Sequencing libraries were prepared using the embryo WGA products and then subjected to detect 24-chromosome copy number variation (CNV) via next-generation sequencing (NGS) according to standard protocol [11]. The excess WGA products were amplified with breakpoint-specific diagnostic primers using PCR for further determined the carrier status of the balanced/euploid embryos and those positive results were predicted to be carrier embryos. Instead, the embryos that showed negative in the breakpoint-specific PCR analyses were noncarrier embryos.

### Prenatal diagnosis

Clinical pregnancy was defined when an intrauterine gestational sac with a heartbeat was observed through ultrasound examination 30–40 days after embryo transfer. Amniocentesis was performed at 18 weeks of gestation, and the amniocentesis fluid sample from fetus was used for karyotyping and SNP-based chromosomal microarray analysis (CMA) analysis to confirm the PGT-SR result. SNP-based CMA using Affymetrix Cytoscan™ 750 K array was performed according to standard protocol.

## Results

Cytogenetic study revealed that wife and husband carried independent balanced reciprocal translocations: 46,XX,t(10;16)(q25.2;q12.1) and 46,XY,t(9;14)(p21.1;q12), respectively (Fig. 1b). The husband’s translocation was familial while the translocation of the wife was de novo. WGLCS technique was subsequently performed on the couple and four derivative fragment sequences (der 10, der 16, der 9 and der 14, respectively) were successfully detected, which identified the breakpoint on chromosome 10 in a 247 bp region at 10q25.2 (chr10: 114078982–114,079,229), the chromosome 16 breakpoint in a 211 bp region at 16q12.2 (chr16: 50898455–50,898,666), chromosome 9 breakpoint in a 1081 bp region at 9q11 (chr9: 27576481–27,577,562) and chromosome 14 breakpoint in a 355 bp region at 14q11.2 (chr14: 26085650–26,086,005) through bioinformatics analysis. The junction fragments were amplified using the junction-spanning primers to confirm the breakpoints, followed by subsequent identification of the precise position of the breakpoints through Sanger sequencing. As shown in Fig. 1c, two accurate translocation breakpoints of the wife were chr10:114079218 & chr16:50898510 and chr16:50898508 & chr10:114079218, respectively; two accurate translocation breakpoints of the husband were chr14:26085924 & chr9:27577048 and chr14: 26085933 & chr9:27577052, respectively. Fortunately, we found that all of the breakpoints were mapped in the intergenic regions, although several nucleotides insertions and/or deletions at the breakpoint junctions in the formation of these two translocations were observed (Fig. 1c).

**Fig. 2:**
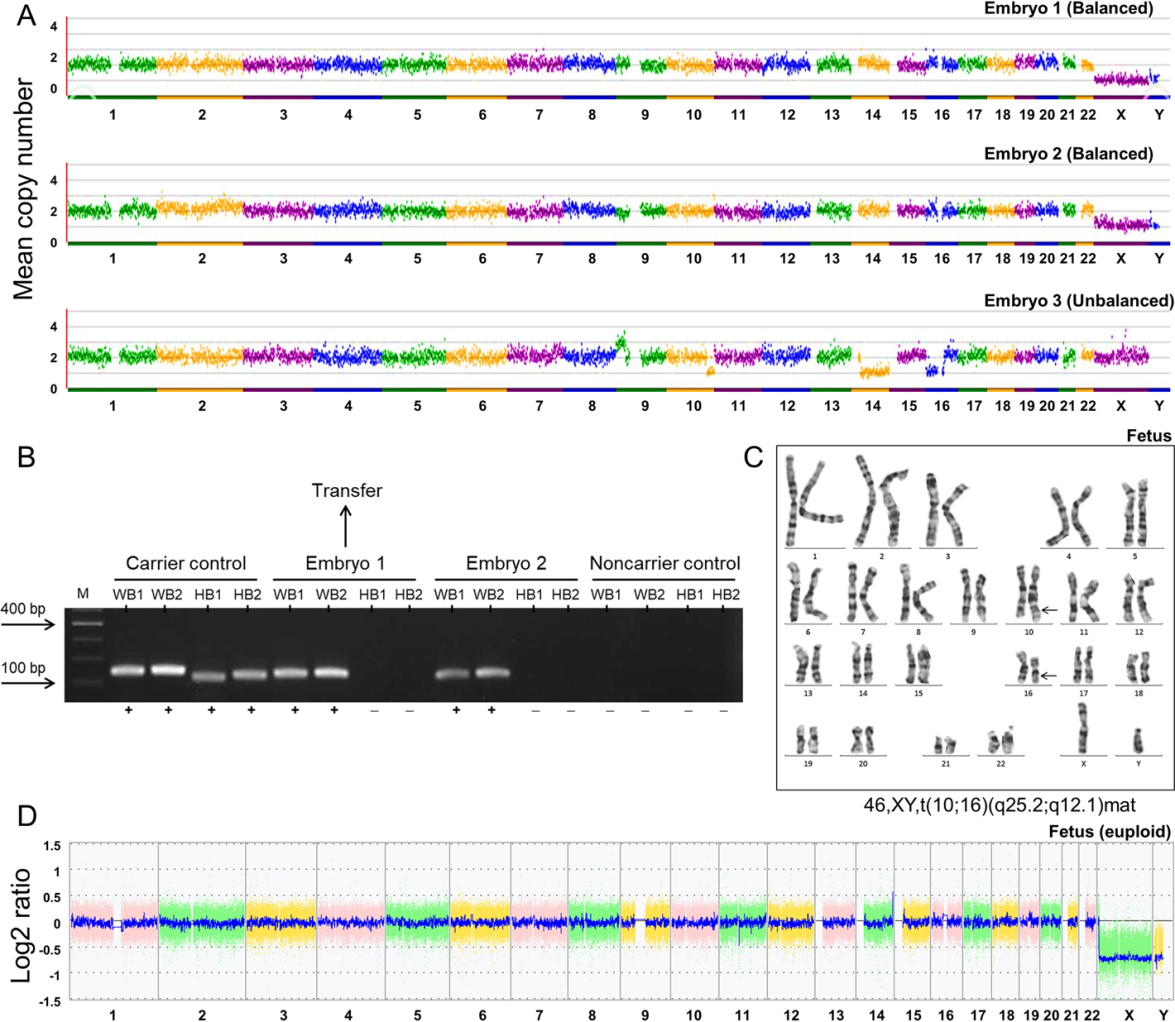
Summary of PGT-SR results of the couple. **a** NGS analysis of three trophectoderm biopsy sample. Embryo 1 and embryo 2 were detected as balanced; Embryo 3 was unbalanced. **b** Diagnosis of the carrier status of embryo 1 and embryo 2. Breakpoint-specific PCR were amplified using the primers WB1-F/R (F: ATTTCTTGGGTGCCCCTCTG, R: AGCATTCTTTCTCACTCATCCCA), WB2-F/R (F: GGGAAATTAGGCAACCCCAAG, R: GAGAGTCTCGCCCAAAGTCA), HB1-F/R (F: CCAATCCGACTGGTTGTGGG, R: TGTGCATGTTAAGCCCACTCT) and HB2-F/R (F: AAGTACACTCCACAGAGTGGG, R: CAGGGAGAGGCAACTTCTCAA); PCR Agarose gel showing the presence (+) or absence (−) of a PCR product for breakpoint or normal sequence; the DNA of the wife and the hasband were uesd for carrier DNA positive controls; a normal DNA was uesd for noncarrier DNA positive control; M = DNA marker; WB1 = wife breakpoint 1; WB2 = wife breakpoint 2; HB1 = husband breakpoint 1; HB2 = husband breakpoint 2. **c** Karyotype of the fetus: 46,XY,t(10;16)(q25.2;q12.1)mat. **d** SNP-based CMA of the fetus

A total of 16 metaphase II (MII) oocytes were retrieved after ovarian stimulation, and 10 were fertilized normally using intracytoplasmic sperm injection (ICSI). At last, three blastocysts (embryo 1, embryo 2 and embryo 3) were subjected to biopsy on day 5 for comprehensive chromosome screening via WGA-based NGS. Unexpectedly, the result indicated that two of them (embryo 1 and embryo 2) were detected as either normal or had balanced translocation from the wife or the husband, and the other one (embryo 3) was unbalanced in all four affected chromosomes: 46,XX,+(9)(p24.3-p21.1)(27.60 Mb),-(10)(q25.3-q26.3)(20.18 Mb),-(14)(q12-q32.33)(91.09 Mb),-(16)(p13.3-q12.1)(49.33 Mb) (Fig. 2a). To distinguish the carrier status of these two balanced/euploid blastocysts (embryo 1 and embryo 2), we then performed breakpoint-specific PCR analyses on the rest of the WGA products. The result showed that both embryo 1 and embryo 2 were carrier embryos with two maternal breakpoints (Fig. 2B), and embryo 1 was transferred on day 5 resulting clinical pregnancy. Cytogenetic analysis was done to confirm the diagnosis at 18 weeks of gestation and revealed the presence of a male karyotype with a heterozygous balanced reciprocal translocation like that present in the mother: 46,XY,t(10,16)(q25.2;q12.1) mat (Fig. 2c). In addition, SNP-array analysis of DNA from amniotic fluid cells demonstrated that the fetus cells were euploid without small segmental chromosome abnormalities (Fig. 2d). A healthy male was born at 40 weeks of gestation by caesarian section. At the time this report was written, the boy was more than 1 year old and showed no malformations and mental retardation.

## Discussion

**Fig. 3:**
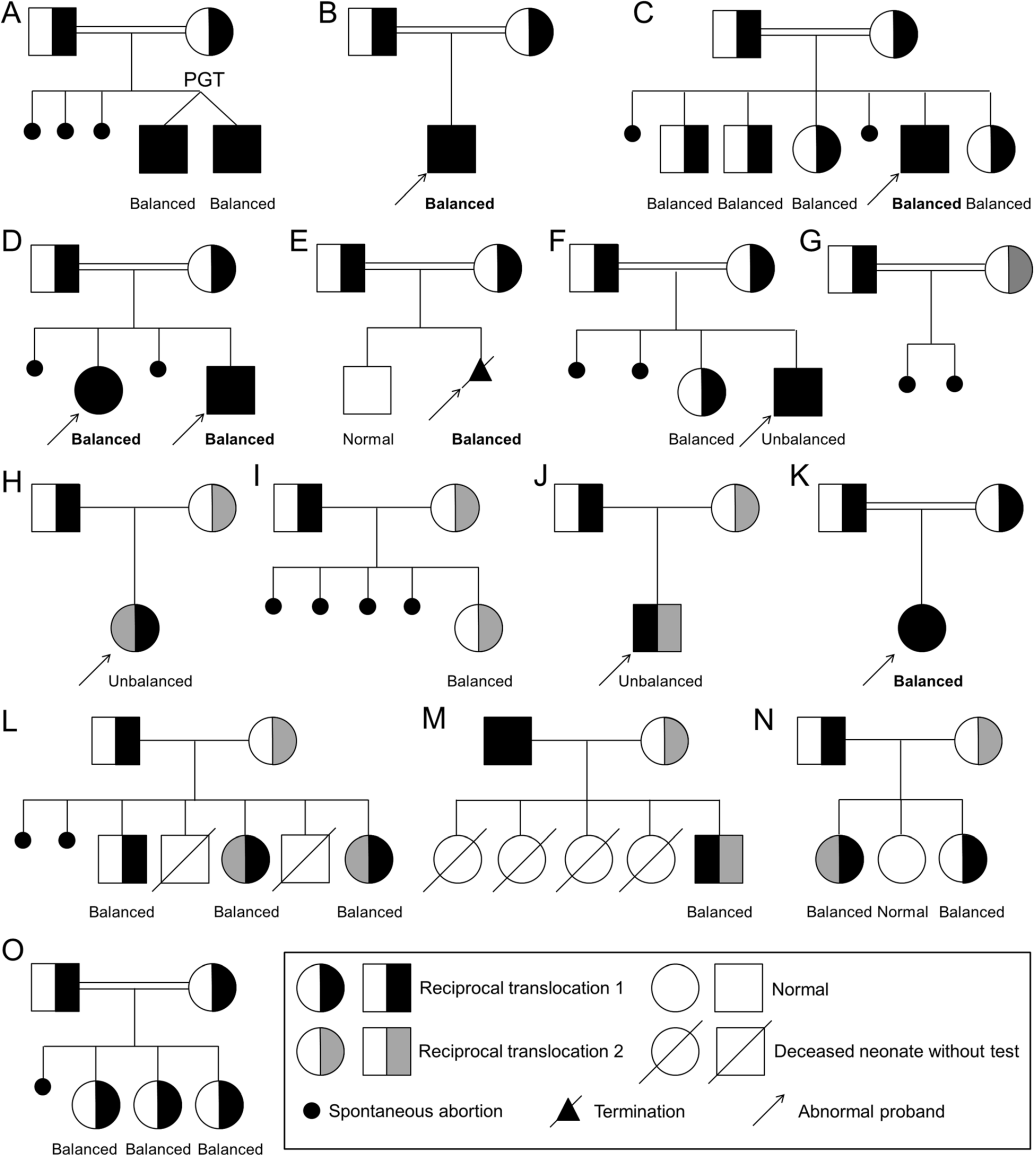
Nuclear pedigrees of couples who both were reciprocal translocation carriers in the literature. Fifty out of 52 recognized pregnancies were natural pregnancies, and the twins of Family A were born through PGT-SR. The karyotypes of the offspring are labeled with “Balanced, Unbalanced or Normal”

The frequency of heterozygous carriers of reciprocal translocations is about 0.16 to 0.2% [1], which means that the probability of two carriers being a couple is less than 4 × 10^− 6^. The case with recurrent miscarriage in the present report represents rare instance in which both parents had an independent balanced reciprocal translocation affecting four chromosomes: 46,XX,t(10;16)(q25.2;q12.2) and 46,XY,t(9;14)(p21.1;q12). Since there are extremely rare PGT-SR data on a couple with two independent reciprocal translocations in reviewing the literature, the reproductive risk assessment and genetic counseling for this case would be unique and complex. However, a considerable number of PGT-SR data with regard to one reciprocal translocation might provide bases for predicting PGT-SR outcomes in couples with two reciprocal translocations. Generally, genetic counseling for a couple with one reciprocal translocation focuses on the risk of unbalanced gametes from one parent. Zhang et al. published a PGT-SR data indicated that the proportion of alternate segregation pattern, which can produce normal/balanced gametes, was 40.7% (749/1842) on average by testing 1842 embryos from 356 carriers of single reciprocal translocations [6]. Given that each partner’s translocation is thought to segregate independently, the risks for generating abnormal gametes might be additive for couples with two reciprocal translocations. Thus, the probability of normal/balanced zygotes for such couples was estimated to be 16.6% (40.7% × 40.7%) without considering non-translocation chromosomes abnormalities. Another large practical PGT-SR data of the European Society of Human Reproduction and Embryology (ESHRE) PGT Consortium showed that 19.5% (4681/23960) day-3 embryos were transferable after genetic testing when one of the partners is a carrier for a reciprocal translocation [8], and this rate increased to 30.0% (142/473) when biopsy at the blastocyst stage [12]. Therefore, theoretically, the transferable embryos rate might be as low as 3.8% (19.5% × 19.5%) in cleavage-stage embryos or 9% (30.0% × 30.0%) in blastocysts for both spouses having a reciprocal translocation. In fact, in 2010 Beyazyurek et al. [13] reported only one PGT-SR study performed for a consanguineous couple carrying the same familial reciprocal translocation between chromosomes 1 and 16 (Table 1 and Fig. 3a), and the result showed that only one out of 15 (6.7%) day-3 embryos was detected as balanced and transferable which is close to the empirical rate that we extrapolated (3.8%). By contrast, surprisingly, we found that 2 out of 3 day-5 blastocysts were balanced for the current couple. The transferable embryo rate was significantly higher than that which would be expected theoretically could be partly explained by chromosome self-correction at the blastocyst stage, as has been suggested by a few authors [28, 29]. The self-correction may occur in a mosaic or aneuploidy embryo. Nevertheless, the probable self-correction of an unbalanced embryo involving translocated chromosome requires further clarification. In fact, it has been reported that the euploidy rate was found to be significantly higher for blastocyst stage embryos as compared to that of cleavage stage embryos (60.3 and 33.4%, respectively) [30]. In addition, another possible explanation is that these two translocations may tend to produce a lower proportion of unbalanced gametes, thus forming a higher proportion of euploidy embryos. Besides, this may due to the small number biopsied blastocysts for PGT-SR, thus more such cases reported and sperm fluorescence in situ hybridization (FISH) analysis [31] would be helpful to predict PGT-SR outcomes. However, other factors including the location of translocation breakpoints, the age of the carriers and chromosome type also made the difference between empirical and practical rates.

**Table 1:** Previously reported couples with two reciprocal translocations

Family	Reference	Karyotypes of couples		Brief clinical details
		Maternal	Paternal	
A	Beyazyurek et al. [13]	46,XX,t(1;16)(q12;q11.2)	46,XY,t(1;16)(q12;q11.2)	3 spontaneous abortions
B	Vu et al. [14]	46,XX,t(16;20)(q21;p11.2)	46,XY,t(16;20)(q21;p11.2)	1 balanced child with phenotypic abnormalities
C	Schneider et al. [15]	46,XX,t(10;11)(q24.3;q23.3)	46,XY,t(10;11)(q24.3;q23.3)	2 spontaneous abortions and 1 balanced child with an abnormal phenotype
D	Zaki et al. [16]	46,XX,t(7;12)(q22;p13)	46,XY,t(7;12)(q22;p13)	2 spontaneous abortions and 2 balanced siblings showed phenotypic abnormalities
E	Martinet et al. [17]	46,XX,t(17;20)(q21.1;p11.21)	46,XY,t(17;20)(q21.1;p11.21)	1 balanced fetus termination with phenotypic abnormalities
F	Kupchik et al. [18]	46,XX,t(16;18)(p13.3;p11.2)	46,XY,t(16;18)(p13.3;p11.2)	2 spontaneous abortions and 1 unbalanced infant with phenotypic abnormalities
G	Ozkul and Dundar [19]	46,XX,t(1;16)(q24;q24)	46,XY,t(1;16)(p22;p13)	2 spontaneous abortions
H	Cook et al. [20]	46,XX,t(2;3)(p13.1;p13)	46,XY,t(7;8)(q36.1;q24.13)	1 unbalanced child with multiple congenital anomalies
I	Tsuji et al. [21]	46,XX,t(7;13)(p15.3;q12.3)	46,XY,t(1;7)(p11.1;q11.1)	4 spontaneous abortions
J	Teivi et al. [22]	46,XX,t(3:4)(p12;15.1)	46,XY,t(5;10)(p12;p13)	1 unbalanced infant with phenotypic abnormalities
K	Wilrnot et al. [23]	46,XX,t(3:16)(p25;q13)	46,XY,t(3:16)(p25;q13)	1 balanced infant with an abnormal phenotype
L	Bowser-Riley et al. [24]	46,XX,t(1;2)(q42:q31.1)	46,XY,(5;19)(p11;q13.1)	2 spontaneous abortions and 2 abnormal deceased neonates
M	Barros et al. [25]	46,XX,t(6;14)(q25;q21)	46,XY,t(1;19)(p11;p11),t(1;19)(p11;p11)	4 previous newborns died in the early neonatal period
N	Mulcahy and Watson [26]	46,XX,t(2;17)(p13;q21)	46,XY,t(1;10)(p34;q24)	Infertility for several years
O	Simoni et al. [27]	46,XX,t(2;7)(p11;q31)	46,XY,t(2;7)(p11;q31)	1 spontaneous abortion

In reviewing the literature, there are 15 couples in which both wife and husband had a balanced reciprocal translocation without clinical expression (Table 1), and Fig. 3 illustrates nuclear pedigrees for them [13–27]: 8 in whom both spouses had an identical balanced reciprocal translocation because of consanguineous marriage (Families A, B, C, D, E, F, K and O), one in whom consanguineous partners were inherited with two similar balanced reciprocal translocations (Family G) and 6 in whom unrelated members of couples carried two different reciprocal translocations (Families H, I, J, L, M and N). Figure 3 shows that there were 52 recognized pregnancies among the 15 couples including 50 natural and 2 PGT-SR pregnancies (Family A), resulting in 19 phenotypically normal live births (2 with a normal karyotype, 11 with a single parental balanced reciprocal translocation and 6 with double parental balanced reciprocal translocations); 14 phenotypically abnormal live births (3 unbalanced offspring, 5 balanced offspring with two identical reciprocal translocations and 6 neonatal deaths without karyotype examination); and 19 abortions (1 termination of pregnancy for an abnormal fetus with two balanced reciprocal translocations and 18 spontaneous abortions). The probability of a clinically recognized pregnancy through natural conception ending with healthy live birth was only 17/50 (34.0%) when both partners carried a balanced reciprocal translocation. However, the overall risk for abnormal live births and abortions/stillbirths in these families was 14/50 (28.0%) and 19/50 (38.0%), respectively. It is worth noting that, 6 offspring were homozygous carriers of translocations in 5 consanguineous couples (marked in bold in Families B, C, D, E, and K). Even though they had apparently balanced karyotypes, multiple abnormal phenotypes were observed. Several studies have suggested that the disease causing genes were disrupted by the breaks and that the affected offspring were homozygous for a recessive gene defect, masked by the unaffected heterozygous parents with a same balanced reciprocal translocation [14–17, 23]. Thus, in this context, the genetic risk of the reciprocal translocations should be specially investigated. It is of great importance to identify whether the translocation breakpoints give rise to gene interruption in PGT-SR treatment and prenatal diagnosis for couples with two balanced reciprocal translocation. The review of the literature indicates that the fetus could indeed inherit unbalanced gametes from mother, father, or both; thus the risk of having abnormal live offspring and of spontaneous abortion might be cumulative in couples with two reciprocal translocations. We believe that PGT-SR would be a useful and practical tool in the aspect of increasing healthy birth rates and decreasing recurrent abortions for such couples.

In recent years, precise translocation breakpoint identification has been increasingly used for estimating the phenotypic outcomes of balanced reciprocal translocations and distinguishing normal and translocation-carrying embryos in PGT-SR cycles. To date, several approaches have been developed to identify transferable translocation-free embryos in PGT-SR treatments, such as mate-pair sequencing [32], MicroSeq-PGD [33], MaReCs [34] and SNP array-based analyses [35, 36]. In this study, we applied WGLCS, an accurate approach which can limit the breakpoints to ±1 Kb region, to initially map the four breakpoint regions of the reciprocal translocations [9]. Then, junction-spanning PCR combined with Sanger sequencing were used to characterize the precise breakpoints. Subsequently, the carrier status of the two balanced/euploid embryos was determined using breakpoint-specific PCR. The sequencing results showed that there were several nucleotides insertions and/or deletions occurred at the breakpoint junctions during the translocation formation. This junction is common in human chromosomal translocations and may arise from a non-homologous end-joining (NHEJ) mechanism [37–39]. Hence, balanced translocations may have imbalances in the molecular level. However, these four breakpoints on chromosome 10, 16, 9 and 14 were mapped in the intergenic regions, which did not cause gene interruption. Therefore, we speculated that no matter their offspring carrying heterozygous balanced reciprocal translocation or double heterozygous balanced reciprocal translocations would have normal phenotype.

## Conclusions

Our study presents a rare example of PGT-SR for reciprocal translocations. To the best of our knowledge, this is the first PGT-SR study performed for a non-consanguineous couple carrying two different reciprocal translocations. The carrier status of the euploid embryos was identified through WGLCS approach combining with breakpoint-specific PCR and Sanger sequencing. The method has potential application in clinical PGT-SR cycles for some patients, particularly those who experienced multiple miscarriages or suffered a clinical phenotype and do not wish to pass on the translocation to their offspring. The healthy live birth in our case and the systematic review of the literature provide a better understanding of reproductive consequences for couples in which both members have a balanced reciprocal translocation and should be useful in PGT-SR, prenatal diagnosis and genetic counseling.

## Data Availability

The data that support the study are available upon reasonable request to the corresponding author.
